# Strategies to Enhance the Data Density in Synchronous Electromagnetic Encoders

**DOI:** 10.3390/s22124356

**Published:** 2022-06-08

**Authors:** Ferran Paredes, Amirhossein Karami-Horestani, Ferran Martín

**Affiliations:** CIMITEC, Departament d’Enginyeria Electrònica, Universitat Autònoma de Barcelona, 08193 Bellaterra, Spain; amirhossein.karami@uab.cat (A.K.-H.); ferran.martin@uab.cat (F.M.)

**Keywords:** electromagnetic encoders, motion sensors, chipless RFID

## Abstract

In this paper, we report two different strategies to enhance the data density in electromagnetic encoders with synchronous reading. One approach uses a periodic chain of rectangular metallic patches (clock chain) that determines the encoder velocity, and dictates the instants of time for retrieving the bits of the identification (ID) code. However, contrary to previous electromagnetic encoders, the ID is inferred at both the rising and the falling edges of the clock signal generated by the clock chain. Moreover, the bits of information are not given by the presence or absence of metallic patches at their predefined positions in the so-called ID code chain. With this novel encoding system, a bit state corresponding to a certain instant of time is identical to the previous bit state, unless there is a change in the envelope function of the ID code signal, determined by the additional non-periodic ID code chain. The other encoding strategy utilizes a single chain of C-shaped resonators, and encoding is achieved by considering four different resonator dimensions, corresponding to four states and, hence, to two bits per resonator of the chain. Thus, with these two strategies, the data density is twice the one achievable in previously reported synchronous electromagnetic encoders.

## 1. Introduction

Electromagnetic encoders constitute an interesting alternative to optical encoders [1,2,3] in many applications devoted to motion control. The most well-known optical encoders are those designated as rotary, consisting of an opaque disc (for example, made of metal) with an array of apertures. The system uses an optical source (that generates an optical beam) and a detector (e.g., a photodiode). When any of the apertures of the encoder lie in the optical path between the source and the detector, the latter detects an optical signal in the form of a pulse. For the determination of the angular velocity, a circular periodic chain of apertures distributed along the rotor perimeter suffices. The time lapse between two adjacent pulses determines the angular velocity, provided the angular distance between consecutive apertures (or period) is well known. The position can be inferred from the cumulative number of pulses retrieved in the detector from a reference position. This is how the so-called incremental encoders work. Nevertheless, in the event of a system reset, incremental encoders cannot provide the angular position. For that purpose, absolute encoders are needed. In such encoders, each position is determined by a unique identification (ID) code, and, for that reason, knowing the previous stages of encoder motion is irrelevant. In optical rotary encoders, the angle resolution is dictated by the number of pulses per revolution (PPR), a figure of merit, and there are commercially available devices with thousands of PPR. However, despite such a very good figure of merit achievable with optical encoders, and the possibility to implement both incremental- and absolute-type encoders, optical signals are affected by dirtiness, grease, or pollution, encountered in many industrial scenarios where encoders operate. Thus, optical encoders offer a limited robustness against hostile surroundings, often present in operational environments. By contrast, microwave signals are more tolerant to pollution and dirtiness. Thus, the microwave counterparts of optical encoders were reported a few years ago in order to alleviate the indicated limitative aspect [4,5,6,7,8,9,10,11]. Such encoders were designated as electromagnetic, or microwave, encoders. Their working principle is similar to the one of optical encoders, the main difference being the fact that, rather than optical signals, electromagnetic encoders use microwaves for the determination of the encoder position and velocity (either linear or angular).

**Figure 1:**
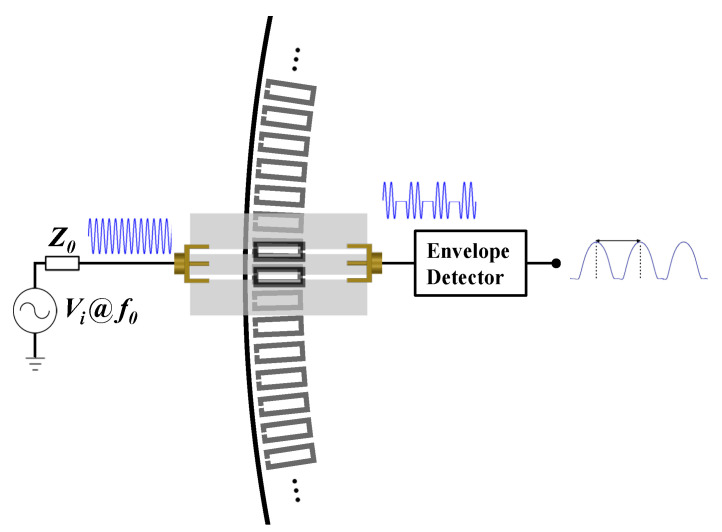
Sketch showing the working principle of an electromagnetic rotary encoder system based on a rotor with a circular chain of split-ring resonators (SRRs).

The first reported electromagnetic encoders were rotary-type and implemented by etching a circular chain of metallic resonators in the periphery of the rotor, a disc made of a dielectric material [4,5]. In the electromagnetic rotary encoder of [5], a figure of merit of PPR = 1200 was demonstrated, and the encoder was equipped with an additional (concentric) non-periodic chain in order to determine the direction of motion, clockwise or counterclockwise. These electromagnetic rotary encoders were incremental. Their working principle was described in [4] (see Figure 1). In brief, a transmission line loaded with a pair of resonators identical to those of the encoder, but oppositely oriented, is the stator, namely, the static part of the system, able to provide the motion variables. When a pair of resonators of the encoder chain crosses the pair of resonators of the stator, the coupling between these elements significantly alters the transmission coefficient of the line. Thus, by feeding the transmission line of the stator with a harmonic signal tuned to a frequency where the transmission coefficient experiences a significant excursion by encoder motion, it is expected that an amplitude modulated (AM) signal is generated at the output port of the line. The envelope function of such AM signal should exhibit as many peaks (or dips) as resonators in the encoder chain (an AM detector suffices to retrieve the envelope function).

Linear electromagnetic encoders based on an identical principle were also reported in several papers [6,7,8,9,10,11]. Indeed, such encoders can be applied not only to the measurement of linear motion (displacement and velocity), but also to the implementation of a kind of time-domain chipless radiofrequency identification (chipless RFID) systems, based on near-field coupling (with the sensitive part of the reader, i.e., a transmission-line-based structure) and sequential bit reading [12,13,14,15,16,17,18]. In this latter application, not all the resonators, or, more generally, the tag inclusions, are present at the periodic positions of the encoder chain. By this means, the encoder, or tag, is provided with a certain ID code, where the logic state ‘1’ or ‘0’ depends on whether a resonator is present or absent, respectively, at its predefined position. A clear advantage of these time-domain near-field chipless RFID systems, as compared to other chipless RFID systems based on time-domain reflectometry (TDR) [19,20,21,22,23,24,25,26,27,28], or as compared to other frequency-domain [29,30,31,32,33,34,35,36,37,38,39,40,41,42,43,44,45,46,47] or hybrid [48,49,50,51,52,53,54,55,56,57,58,59,60,61] chipless RFID systems, is the fact that the number of bits is only limited by tag size. Moreover, the tags are read sequentially, and a simple harmonic signal suffices for tag reading (by contrast, in frequency-domain and hybrid chipless RFID systems, wideband signals for tag reading are needed).

Rigorously speaking, in their functionality as motion sensors, linear or rotary electromagnetic encoders able to provide the absolute position have not been reported so far. The main limitation concerns the fact that providing an ID code identifying the different positions, given by the ratio between the encoder length (in linear encoders), *L*, and the encoder period, *p*, may represent an excessive number of bits. For example, to unequivocally determine the different positions in a linear encoder with *L* = 1 m, and *p* = 1 mm, the number of required bits, *N*, must satisfy.

$$N\geq \log_{2}\frac{L}{p}\;\left(1\right)$$

i.e., in the considered example, the minimum number of bits is *N* = 10. However, it is not possible to consider an encoder implementation with 10 parallel chains of inclusions (i.e., with as many chains as required number of bits). The solution was reported in [8] and referred to as a quasi-absolute electromagnetic encoder.

In the present paper, one objective is to briefly review the working principle of quasi-absolute electromagnetic encoders based on synchronous reading, and provide a prototype example (this is the subject of Section 2). Then, in Section 3, two different strategies to enhance the data density in electromagnetic encoders with synchronous reading are discussed and validated by means of two prototype examples. This is the main motivation of this paper, as far as these new encoders with enhanced data density represent a clear advance as compared to previous synchronous electromagnetic encoders, mainly reported in [8,9,10,11].

## 2. Quasi-Absolute Electromagnetic Encoders

A quasi-absolute electromagnetic encoder is a system where the encoder is able to provide the velocity and the absolute position (by contrast, in incremental encoders, the cumulative number of pulses from a reference position gives the encoder location). However, after a system reset, the quasi-absolute encoder must move several periods in order to be able to provide the encoder position. Such number of periods is given by the number of bits, *N*, that are necessary to univocally determine all the different positions of the encoder and given by expression (1). Thus, because the absolute position cannot be immediately determined after a system reset, such encoders have been designated as quasi-absolute encoders. The whole encoder must be codified following the so-called De Bruijn sequence [62], where any subset of *N* consecutive bits does not repeat along the length of the encoder. By this means, it is guaranteed that all the different encoder positions are identified by a different (and unique) ID code. In the first quasi-absolute electromagnetic encoders reported in the literature, the clock signal, which provides the encoder velocity, determines the instants of time for reading a unique bit of the ID code. The encoder is based on two parallel chains, the clock chain and the ID code chain. The clock chain is periodic, i.e., all the considered inclusions are present at their predefined positions. By contrast, in the ID code chain, only those inclusions corresponding to the logic state ‘1′ are present (i.e., the ID code chain is non-periodic). Various realizations of quasi-absolute electromagnetic encoders have been recently reported [8,10,11]. In this paper, we report the one presented in [11], in order to compare it with the implementations based on strategies that double the number of bits per clock pulse, to be discussed in the next section, and representing the relevant contribution of the present paper.

**Figure 2:**
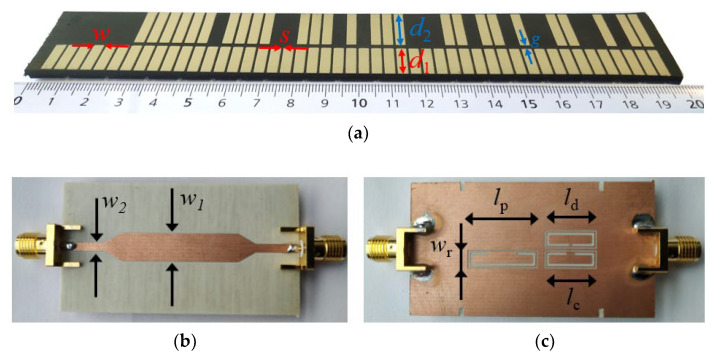
Photograph of the quasi-absolute electromagnetic encoder implemented on a rubber elevator belt (**a**) and photograph of the top (**b**) and bottom (**c**) views of the reader. The dimensions of the encoder are: *d*_1_ = 11.5 mm; *d*_2_ = 15.9 mm, *w* = 3 mm, *s* = 1 mm and *g* = 1.9 mm. The chain period is *p* = 4 mm. Note that *L*/*p* = 48; thus, *N* = 6. The reader (stator) was fabricated on the *Rogers RO4003C* substrate with thickness *h* = 0.81 mm, dielectric constant *ε*_r_ = 3.38, and loss factor tanδ = 0.0027. The dimensions of the stator are: transmission line widths *w_1_* = 6.4 mm and *w_2_* = 1.9 mm; CSRR widths *w_r_* = 2.9 mm (for the three resonators); resonator lengths *l_c_* = *l_d_* = 10.5 mm and *l_p_* = 14.5 mm; ring splits *s_d_* = 0.4 mm, *s_c_* = 1.6 mm, and *s_p_* = 6.2 mm; CSRR slot width *c* = 0.5 mm. The sub-index c, d, and p in the variables corresponding to resonator’s length and ring splits are used to differentiate the CSRRs.

Figure 2 depicts the photograph of the quasi-absolute encoder of [11], implemented in a rubber elevator belt. The clock and ID chains are based on rectangular patch inclusions. Such inclusions can be detected by means of a transmission (microstrip) line loaded with a complementary split-ring resonator (CSRR) [63], etched in the ground plane, beneath the line. The reason is that the presence of a metal patch in close proximity to the CSRR modifies the frequency response (transmission coefficient) of the line. Particularly, the resonance frequency shifts up. Therefore, by injecting a harmonic signal tuned to the resonance frequency of the bare CSRR, a significant excursion of the transmission coefficient at that frequency is expected when a metal patch crosses the area of influence of the CSRR. Consequently, an amplitude-modulated (AM) signal generated by encoder motion is expected. For the periodic clock chain, the envelope function of this AM signal should be periodic. However, in the considered encoder reader, also depicted in Figure 2, three different CSRRs can be appreciated in the ground plane. One of these CSRRs, the one designated as CSRR_p_, is devoted to detecting the inclusions of the ID code chain, another one (CSRR_c_) is used to detect the inclusions of the clock chain, and, finally, it can be appreciated that there is a third resonant element (CSRR_d_) that lies in the path of the clock chain, but not centered beneath the line. This latter CSRR_d_ is used to generate a redundant clock signal with lag or lead as compared to the original one. By this means, it is possible to determine the direction of motion. Obviously, three harmonic signals tuned to the resonance frequency of the three CSRRs are needed. In order to retrieve the three envelope functions in a real system, one option is to feed the line by means of the three harmonic signals using a combiner, and then separate the AM signals at the output port by means of a triplexer. By connecting an envelope detector to each of the output ports of the triplexer, the three envelope functions can be inferred. An alternative is to use a voltage-controlled oscillator (VCO) managed by a microcontroller, in order to provide the three harmonic signals consecutively during different periods of time (the solution adopted in [11]). The microcontroller separates also the three envelope functions corresponding to the clock, redundant and ID code signals (see further details in [11]).

**Figure 3:**
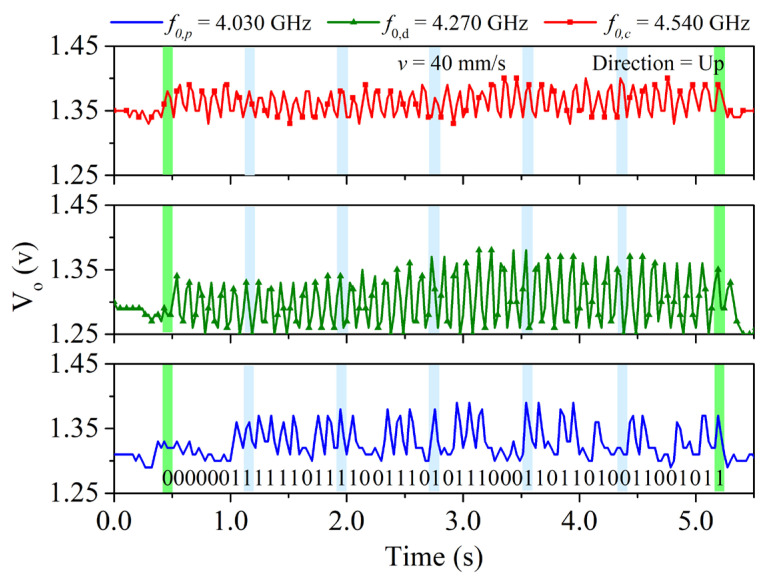
Measured envelope function for the clock, direction, and position signals corresponding to the quasi-absolute encoder system of Figure 2. Reprinted with permission from [11].

In order to demonstrate the functionality of the encoder, it was displaced at a velocity of *v* = 40 mm/s in [11] by means of a dedicated experimental setup based on a linear displacement system (model *Thorlabs LTS300/M* (Thorlabs Inc., Newton, NJ, USA)). The air gap, or separation between the stator and encoder, was set to 1 mm. The *HMC391LP4* VCO (Analog Devices, Norwood, MA, USA) was used for the harmonic signal generation. The control voltage of this component was managed by means of the *ATmega328* microcontroller (Atmel Corporation, San Jose, CA, USA) (*Arduino* development platform), whereas the considered envelope detector was the *ADL5511* model (further details are given in [11]). Figure 3 shows the three envelope functions, after being retrieved by the microcontroller. The clock and the ID code signals exhibit good synchronism, whereas the direction signal is delayed with regard to the clock signal, thus indicating that the encoder moves upwards (in reference to Figure 2c). The separation between peaks is 100 ms, which corresponds to an encoder velocity of 40 mm/s, since the period of the metallic inclusions (resolution) is *p* = 4 mm. Note also that the peaks in the ID code signal perfectly correlate with the presence of rectangular patches in the position chain (see Figure 2). Let us clarify that the maximum velocity of these synchronous encoder systems is determined by the sampling period of the microcontroller, as discussed in [11]. In the systems of [11], the maximum velocity was estimated to be roughly 100 mm/s. Nevertheless, with certain microcontrollers the sampling period can be reduced to a few μs, and encoder velocities in the range of several meters per second can thus be measured.

## 3. Enhancing the Data Density

In this section, two different strategies to enhance the data density per unit length in quasi-absolute electromagnetic encoders are reported. With both approaches, the density of bits per unit length is doubled, which means that, after a system reset, the necessary number of periods that the encoder should displace in order to provide the absolute position is half the number of periods in the electromagnetic encoder reported in the previous section. Let us next discuss both strategies separately.

### 3.1. Encoder Reading by Rising/Falling Edges of the Clock Signal

**Figure 4:**
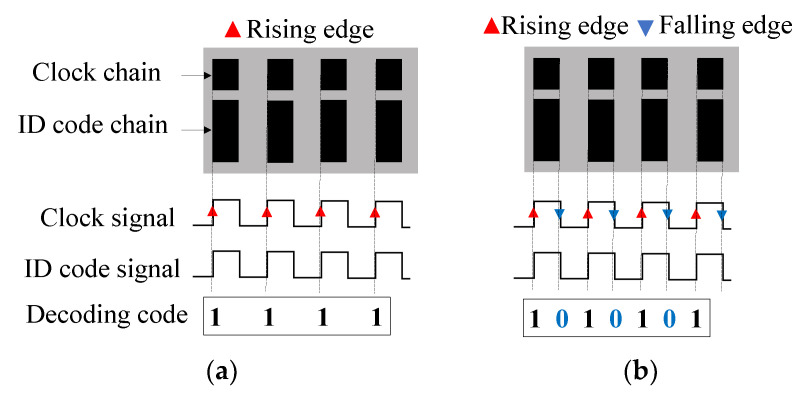
Scheme of the encoding system based on the rising (**a**) and rising/falling (**b**) edge of the clock signal. Reprinted with permission from [64].

In the quasi-absolute encoder of the previous section, the ‘0’ and ‘1’ logic states are given by the absence and presence, respectively, of metallic inclusions (rectangular metallic patches) at the predefined positions in the ID code chain. In that system, the clock signal dictates the instants of time where the bits of the encoder (ID code chain) must be read. Typically, these instants of time are the rising edges of the digital clock signal. A novel encoding strategy, useful to increase the data capacity using the same encoder area, is illustrated in Figure 4 [64]. Compared to the strategy of the encoder of Figure 2, with this novel approach, the rising and the falling edges of the digital clock signal dictate the instants of time for synchronously reading the bits of the encoder. This means that the sampling rate of the ID code signal increases by a factor of two, as compared to the conventional encoder, where the bits of the ID code chain are retrieved at the rising edges of the clock signal. With this strategy, the data density per unit length is enhanced approximately by a factor of two (2N-1). An important feature of this novel encoding scheme is that the bit state corresponding to a certain instant of time is identical to the previous bit state, unless there is a change in the envelope function of the ID code signal. This means that encoding is not based on the absence or presence of functional inclusions at their predefined positions (the procedure of the encoder of Section 2). By contrast, with this encoding scheme, in the rising and falling edges of the clock signal, the new bit is identical to the previous one, if the envelope function of the ID code chain does not change, and the new bit switches provided the envelope function experiences a variation. Thus, the encoders exhibit ID code chains, where, rather than all-identical metallic patches at predefined positions, they are made, in general, of unequal patches, with size dependent on the number of consecutive ‘1’ in the ID code chain. To clarify this aspect, note that the first ‘1’ of the ID code chain should correspond to the presence of metal in that chain (i.e., the envelope function up) for the reading edge (either rising or falling) of the clock chain.

**Figure 5:**
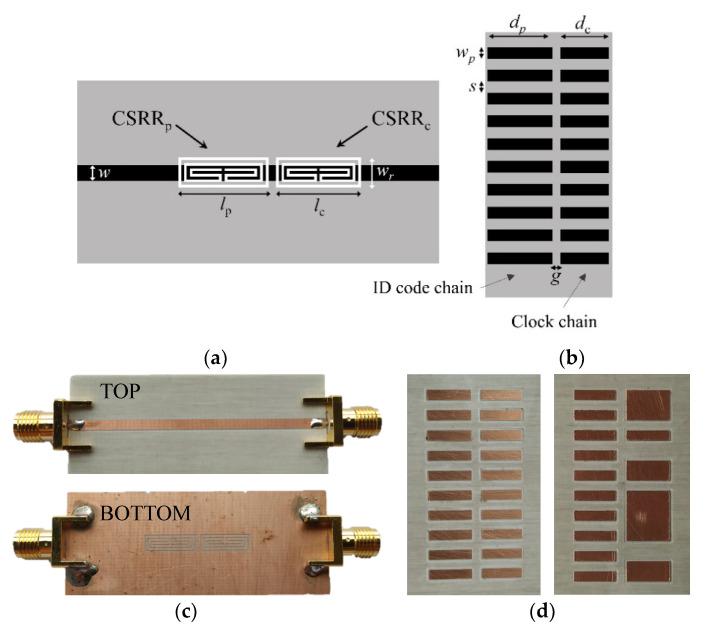
Layout of the reader (stator) (**a**) and specific encoder (**b**), and photograph of the reader (**c**) and two specific encoders (**d**). Dimensions (in mm) are: *w* = 1.90, *w_r_* = 3.30, *l_p_* = 9.35, *l_c_* = 9.90, *d_p_* = 8.15, *d_c_* = 8.70, *w_p_* = 2.00, *s* = 2.00, and *g* = 2.00. The considered substrate for the reader is the Rogers RO4003C with relative permittivity *ε_r_* = 3.55, thickness *h* = 0.81 mm, and loss tangent tanδ = 0.0021. For the encoders, the substrate is the same as the one used in the reader, but with thickness *h* = 0.204 mm. Reprinted with permission from [64].

For validation of this approach, a reader (stator) similar to the one of the system in Section 2 was fabricated (see Figure 5). The main difference is that in this new reader, only two CSRRs are present, namely, the encoder does not have the possibility to determine the motion direction. The encoders are based on two chains of inclusions, the clock chain, identical to the one of the encoder in Figure 2a (although the dimensions are somehow different), and the ID code chain. Figure 5 also depicts two 19-bit specific encoders.

**Figure 6:**
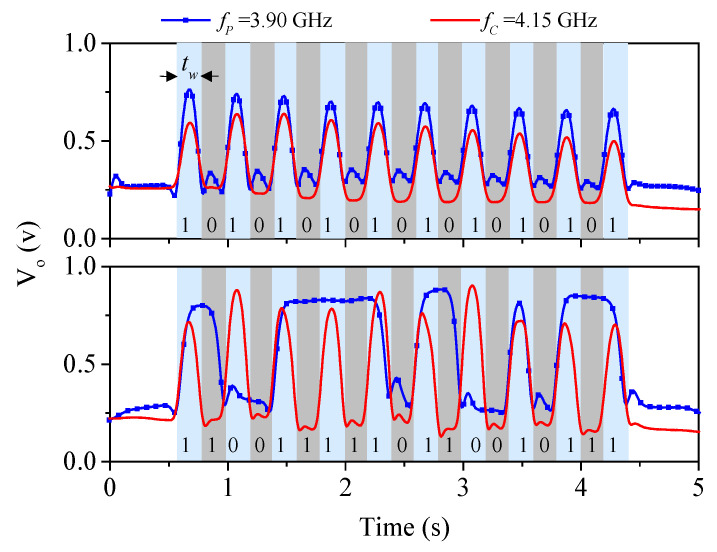
Measured envelope signals (ID code and clock) of the 19-bit tags of Figure 5 with the indicated codes. Reprinted with permission from [64].

To determine with precision the frequency of the interrogation signals, the frequency response of the reader, without rectangular metallic patches on top of it, was measured. The results (not shown) indicate that the notches appear at f_c_ = 3.90 GHz and f_p_ = 4.15 GHz, and these should be the frequencies of the ID code and clock signals, respectively. The experimental setup for characterization of this new encoder system uses also a linear displacement system identical to the one used for the characterization of the encoder system in Section 2. By considering a nominal air gap of 0.5 mm, the measured envelope signals (ID code and clock) of the two 19-bit encoders have been found to be those depicted in Figure 6. Such signals perfectly correlate with the encoders of Figure 5. Note that the sampling rate, as indicated, is twice the one of the system in Section 2; therefore, the data density is twice the one of that system. Thus, the number of periods of the encoder that are necessary to retrieve the encoder position after a system reset is reduced to half the value of the system based on the encoding scheme reported in Section 2.

### 3.2. Hybrid Time-/Frequency-Domain System

According to this encoding strategy, the proposed system is a hybrid time-/frequency-domain encoder system [65]. The encoders consist of a single chain of C-shaped resonators etched in a dielectric substrate. The bits of the encoder are read sequentially through the near field, similar to the previous encoder systems, in a time-division multiplexing scheme. However, there is a fundamental difference between the system to be discussed in this subsection and those analyzed before in this paper. That is, in the present system, there is always a C-shaped resonator in the predefined (periodic) positions of the chain. Thus, an additional chain to provide synchronism (clock chain) is not necessary. Thus, encoding is not achieved by the presence or absence of inclusions (C-shaped resonators), but through the size of such resonators. Four different sizes are considered, corresponding to four different states and, consequently, to two bits per resonant element of the chain. Because the size of the resonator correlates with its resonance frequency, it follows that bit encoding is achieved by frequency. For this reason, this system is designated as a hybrid time-/frequency-domain encoder system. The information is encoded in frequency, but it is read sequentially in time domain. However, contrary to the system in Section 2, in the present system, each reading (detection of the size of the C-shaped resonator) provides two bits of information rather than one. Thus, with this system, the data density is twice the one of those systems based on presence/absence of inclusion at the predefined positions in the encoder chain.

**Figure 7:**
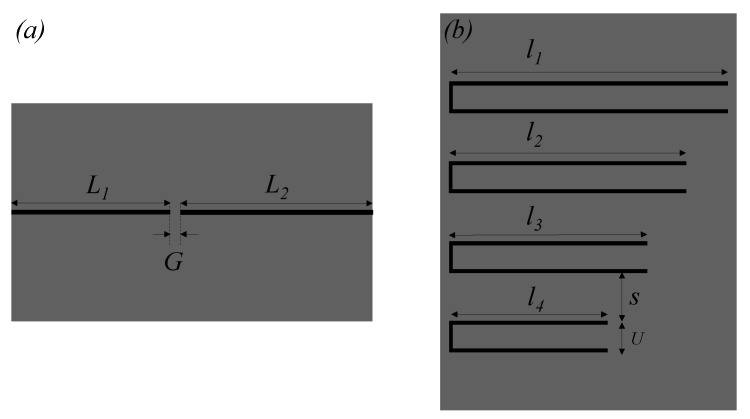
Topology of the (**a**) reader (sensitive part) and (**b**) 8-bit (or 4-resonator) encoder.

The sensitive part of the reader (stator) is a transmission line with a series gap, whereas the tag is a chain of C-shaped resonators, as indicated. Figure 7 depicts the layout of the reader (sensitive part), as well as the one of a specific tag made of four C-shaped resonators (i.e., an 8-bit tag), all exhibiting different size. The symmetry plane of the C-shaped resonators is an electric wall at the first (fundamental) resonance, and a magnetic wall at the second resonance frequency. Thus, by displacing the tag across the line, the coupling between the resonator and the line arises at the second resonance frequency, rather than at the fundamental one, when one of the resonant elements crosses the line axis. The reason is that the quasi-microstrip mode of the line is an even mode, unable to drive the odd mode resonance of the C-shaped resonators. Due to the presence of the gap, the structure exhibits a bandpass behavior in the vicinity of the second resonance frequency of the C-shaped resonator on top of the line. Thus, by feeding the gap-loaded line with four harmonic signals tuned to the second resonance frequency of each resonator, the ID code can be retrieved.

For each harmonic signal, tag displacement on top of the reader generates an amplitude-modulated (AM) signal. The amplitude of the envelope function should be negligible unless a C-shaped resonator tuned (second resonance) to the frequency of that harmonic signal is on top of the line. Thus, by recording the envelope function for each harmonic signal, the ID code can be obtained. An input combiner can be used to feed the reader line with the four harmonic signals, and an output multiplexer can be used for the separation of the different AM signals. In this case, four envelope detectors are needed. Thus, a preferred option should consider a voltage-controlled oscillator (VCO) managed by a microcontroller, so that the frequency of the single injected signal varies periodically between the four required values (second resonance frequencies of the C-shaped resonators). The AM signals are separated at the output port also by means of the microcontroller; thus, the four envelope functions can be inferred. With this approach, a single envelope detector is needed.

The line of the reader was implemented in the *Rogers RO4003C* substrate with dielectric constant *ε_r_* = 3.55, thickness *h* = 0.81 mm, and loss factor tanδ = 0.0022. The width of the line is *w* = 0.40 mm, gap separation is set to *G* = 1 mm, and the total line length from port to port is *L* = 50 mm. The gap is not in the middle of the microstrip line, as it is shown in Figure 7, where *L*_1_ = 22 mm and *L*_2_ = 27 mm. The tags were implemented in the *Rogers RO4003C* substrate with dielectric constant *ε_r_* = 3.55, thickness *h* = 0.81 mm, and loss factor tanδ = 0.0022. The strip width of the C-shaped resonators is 0.25 mm, whereas the length varies from resonator to resonator. Indeed, only the length of the external arms varies, i.e., it is *l*_4_ = 16 mm, *l*_3_ = 20 mm, *l*_2_ = 24 mm, and *l*_1_ = 28 mm from the smallest to the largest resonator. The length of the central strip of the C-shaped resonators is *U* = 3 mm in all cases, and the separation between adjacent resonators is *S* = 5 mm, corresponding to a period of 8 mm. The (second) resonance frequencies of these resonators are *f*_0,1_ = 3.70 GHz, *f*_0,2_ = 4.25 GHz, *f*_0,3_ = 5.00 GHz, and *f*_0,4_ = 5.80 GHz.

**Figure 8:**
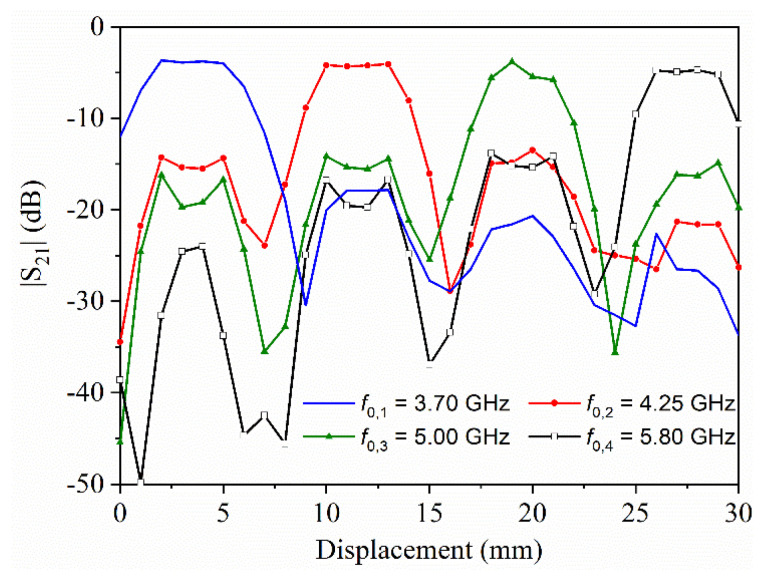
Simulation of the transmission coefficient for the resonance frequencies of the four C-shaped resonators of the encoder, as a function of the encoder displacement. The considered encoder is depicted in Figure 7. Reprinted with permission from [65].

Before the characterization of this type of encoders, we carried out a set of simulations, consisting of obtaining the transmission coefficient as a function of the displacement for a tag based on four resonators, each with different dimensions. This simulation, carried out by means of the *ANSYS HFSS* commercial software, was obtained four times, each one by considering the (second) resonance of each resonator (the results are depicted in Figure 8). In all the simulations, the air gap was set to 0.5 mm, and the displacement step was set to 1 mm which is several times smaller than the chain period. With this displacement step, the accuracy in the response is good enough to conclude that the capacity of discrimination of the proposed system is very reasonable. In particular, it can be appreciated that when a certain resonator is on top of the line, at the frequency of this resonator, the transmission is high, whereas at the other frequencies, it decreases significantly, and this occurs for the four C-shaped resonators. Namely, the excursion (or window) between the high and low levels of transmission is important, a necessary condition for the discrimination and functionality of the system. Thus, these results indicate that the functionality of this encoding near-field chipless RFID system can potentially be achieved.

**Figure 9:**
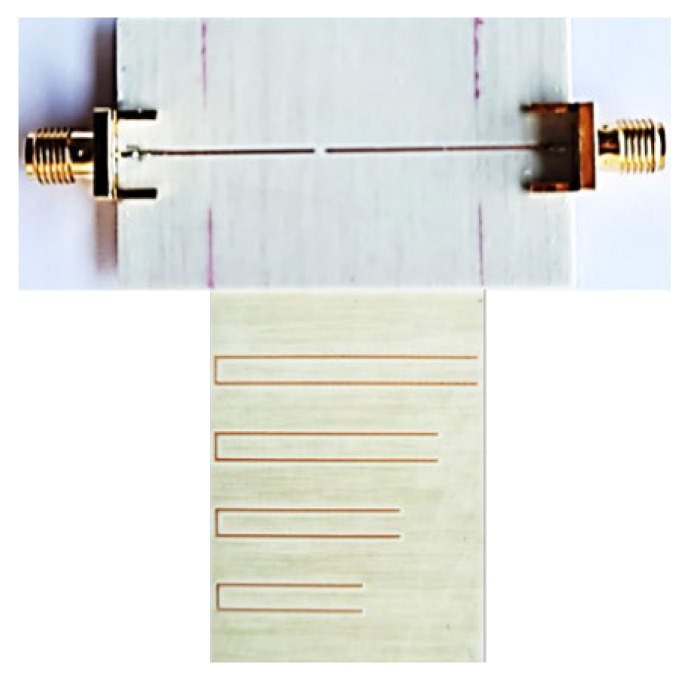
Photograph of the fabricated reader and encoder. Reprinted with permission from [65].

**Figure 10:**
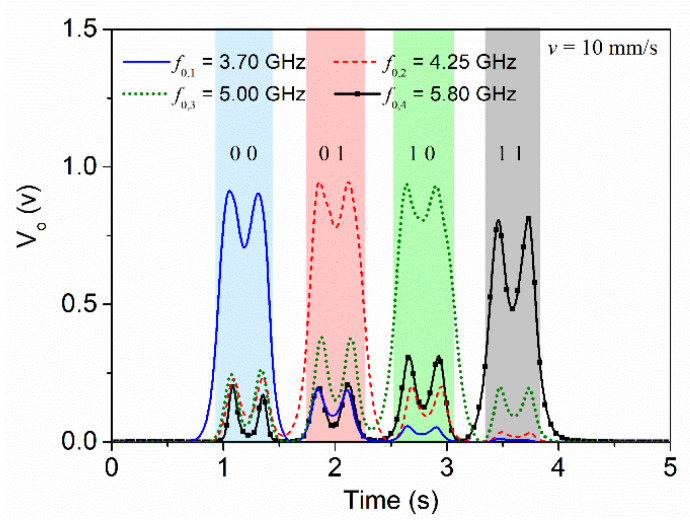
Envelope functions for the four frequencies corresponding to the (second) resonances of the four C-shaped resonators, for the tag depicted in Figure 9. Reprinted with permission from [65].

For experimental validation, the reader and the encoder depicted in Figure 7 were fabricated. The photograph of the fabricated reader and tag are shown in Figure 9. For tag reading, we displaced the tag over the reader by means of a linear displacement system previously indicated with a velocity of 10 mm/s (the air gap was set to 0.5 mm). The four envelope functions recorded for each frequency, when the tag is displaced across the line axis of the reader, are depicted in Figure 10. With these results, the proof-of-concept of the proposed system is experimentally validated, since each resonator is perfectly identified. Note that for each carrier frequency, a different peak in the envelope function arises, indicative of the fact that the tag is made of four different resonators.

**Figure 11:**
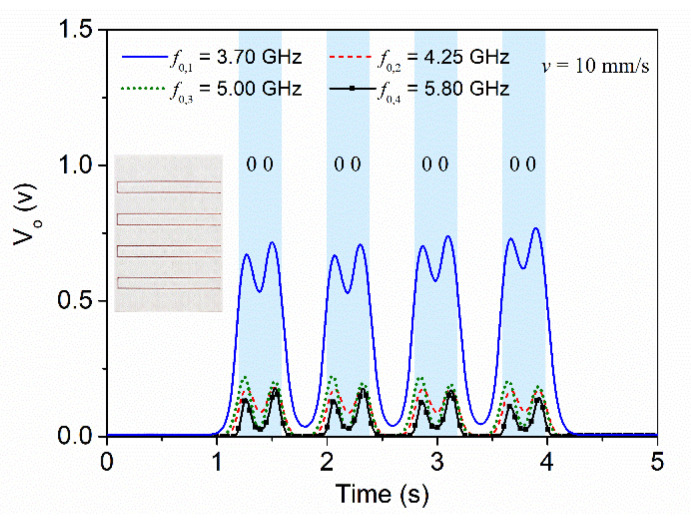
Envelope functions for the four frequencies corresponding to the (second) resonances of the four C-shaped resonators, for the tag depicted in the inset. Reprinted with permission from [65].

To further demonstrate the functionality of this system, we fabricated additional encoders. For example, an encoder where all the C-shaped resonators are identical, and corresponding to the largest one, is depicted in Figure 11, where the envelope functions are also shown. It can be observed that the responses perfectly correlate with the encoder of the inset.

**Figure 12:**
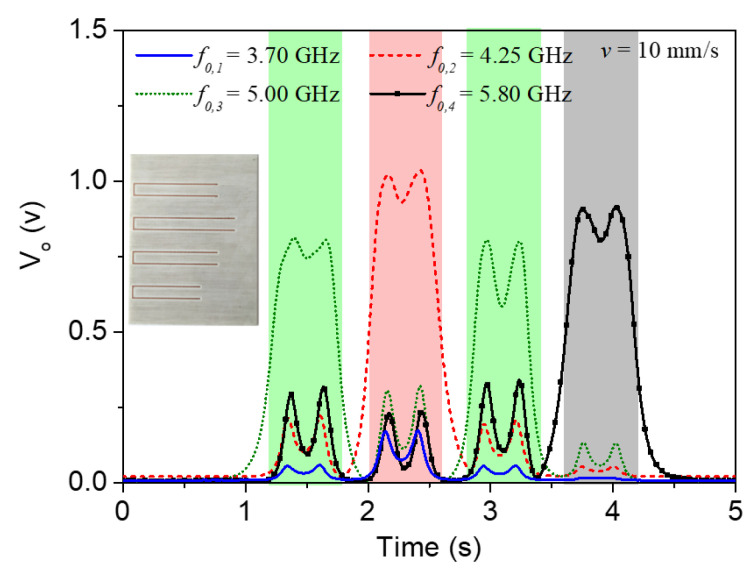
Envelope functions for the four frequencies corresponding to the (second) resonances of the four C-shaped resonators, for the tag depicted in the inset.

Figure 12 depicts a different combination of resonators and the corresponding envelope functions where, again, there is a perfect correlation between the peaks and the resonant elements.

**Figure 13:**
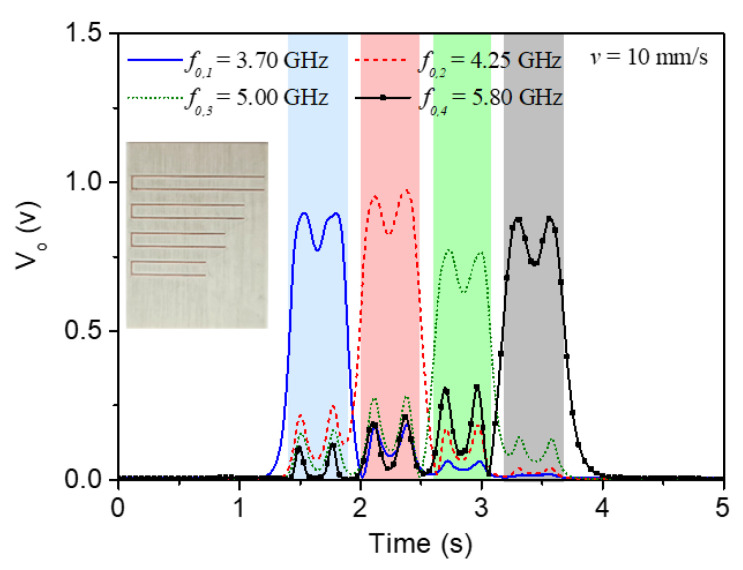
Envelope functions for the four frequencies corresponding to the (second) resonances of the four C-shaped resonators, for the tag depicted in the inset.

We have also fabricated encoders with reduced period, particularly by reducing the distance between adjacent C-shaped resonators. Thus, Figure 13 depicts a 6 mm period encoder where the four resonators are different, and the corresponding envelope functions. Note that the envelope function responses are similar to those of Figure 10, where identical resonators are considered. However, in Figure 13, the time interval between the first and the last pulse is smaller by virtue of the smaller period in the structure of Figure 13 (the displacement velocity of the encoder is identical in all the cases).

**Figure 14:**
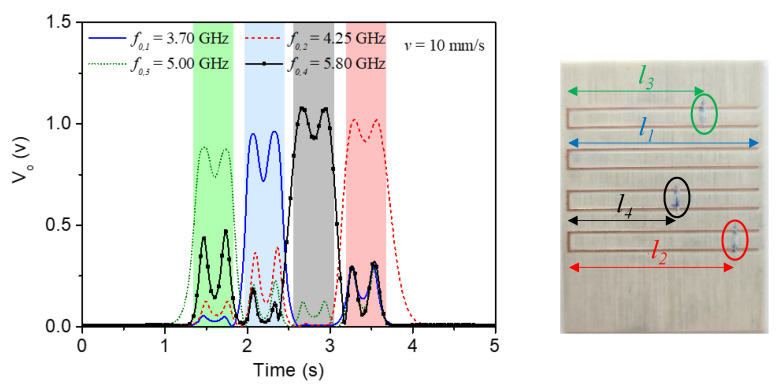
Envelope functions for the four frequencies corresponding to the (second) resonances of the four C-shaped resonators, for the indicated encoder, with cuts in certain resonators. The lengths of the resonators, after practicing the cuts, correspond to those lengths shown in Figure 7b.

Finally, let us mention that it is possible to program the encoders. That is, by fabricating the encoders with all the resonators identical and corresponding to the largest one, it is possible to generate the desired code by simply cutting the convenient resonators. For example, it can be observed in Figure 14 an encoder where three of the C-shaped resonators are cut By this means, four different resonances are generated, as can be seen in the envelope functions, equivalent to an encoder exhibiting four different resonators. Note, however, that the bit combination is different to that of Figure 13, since the order of the resonators in the encoder is also different.

## 4. Discussion

The quasi-absolute electromagnetic encoders reported in this paper cannot compete against the absolute optical encoders in terms of resolution. However, electromagnetic encoders use microwave signals, more robust against the effects of pollution, grease, or dirtiness encountered in many industrial scenarios. Let us also mention that there are many displacement and proximity sensors based on the Hall effect [66,67,68,69,70,71,72]. Although these systems generally exhibit very good resolutions, they are based on magnets, thereby representing solutions with higher cost, at least compared to the systems reported in this paper. Magnetic encoders have also been reported [73,74]. Such encoders are also robust against hostile environments, but such sensors are relatively complex since they need inductive coils.

Concerning other displacement, proximity, and velocity sensors based on microwave signals, there are many implementations in the available literature. For example, there are sensors that exploit coupling modulation [75,76,77,78,79], whereas other sensors are based on frequency variation [80]. In such sensors, a resonant (movable) element is displaced over a transmission line structure (typically, the resonator in motion is displaced in a plane parallel to the one of the line, but there are also realizations, where the resonator displaces vertically [79,81]). Although these sensors are relatively simple, their input dynamic range is usually very limited, since it is of the order of the size of the resonant elements. By contrast, in linear electromagnetic encoders, such dynamic range is potentially unlimited. Thus, electromagnetic encoders can find applications as sensing elements in various scenarios involving motion control of industrial systems, such as conveyor belts, servomechanisms, or elevators, among others. It is also worth mentioning that there are alternatives to the electromagnetic rotary encoders, based on the use of a single resonant element [82,83,84]. Generally, such angular displacement and velocity sensors use a circular resonator axial to the rotor. The main disadvantage as compared to the rotary electromagnetic encoders is the fact that they cannot provide the instantaneous velocity, but rather the average velocity per cycle.

## 5. Conclusions

In summary, two different strategies to enhance the data density in electromagnetic encoders were reported. Such strategies are very different, but both share the fact that the data density is twice the one of conventional encoders. In one case, the data density is improved by retrieving the ID code in both the rising and falling edges of the clock signal. Namely, this system requires a clock signal in order to synchronously read the ID code. By contrast, in the second approach, such a clock signal is not required, as far as an inclusion is always present at the predefined positions of the encoder chain. In this second approach, the data density is improved because each resonant element of the encoder chain provides two bits of information, as far as four different resonator sizes are considered. Both strategies have been experimentally validated by considering various encoders with different ID codes. The reported approaches are of interest for the implementation of quasi-absolute electromagnetic encoders. Thanks to the improved data density, the displacement of the encoder necessary to retrieve the absolute position after a system reset can be reduced.
